# Classification of Children With Autism and Typical Development Using Eye-Tracking Data From Face-to-Face Conversations: Machine Learning Model Development and Performance Evaluation

**DOI:** 10.2196/29328

**Published:** 2021-08-26

**Authors:** Zhong Zhao, Haiming Tang, Xiaobin Zhang, Xingda Qu, Xinyao Hu, Jianping Lu

**Affiliations:** 1 Institute of Human Factors and Ergonomics College of Mechatronics and Control Engineering Shenzhen University Shenzhen China; 2 Shenzhen Guangming District Center for Disease Control and Prevention Shenzhen China; 3 Department of Child Psychiatry of Shenzhen Kangning Hospital Shenzhen Mental Health Center Shenzhen China

**Keywords:** autism spectrum disorder, eye tracking, face-to-face interaction, machine learning, visual fixation

## Abstract

**Background:**

Previous studies have shown promising results in identifying individuals with autism spectrum disorder (ASD) by applying machine learning (ML) to eye-tracking data collected while participants viewed varying images (ie, pictures, videos, and web pages). Although gaze behavior is known to differ between face-to-face interaction and image-viewing tasks, no study has investigated whether eye-tracking data from face-to-face conversations can also accurately identify individuals with ASD.

**Objective:**

The objective of this study was to examine whether eye-tracking data from face-to-face conversations could classify children with ASD and typical development (TD). We further investigated whether combining features on visual fixation and length of conversation would achieve better classification performance.

**Methods:**

Eye tracking was performed on children with ASD and TD while they were engaged in face-to-face conversations (including 4 conversational sessions) with an interviewer. By implementing forward feature selection, four ML classifiers were used to determine the maximum classification accuracy and the corresponding features: support vector machine (SVM), linear discriminant analysis, decision tree, and random forest.

**Results:**

A maximum classification accuracy of 92.31% was achieved with the SVM classifier by combining features on both visual fixation and session length. The classification accuracy of combined features was higher than that obtained using visual fixation features (maximum classification accuracy 84.62%) or session length (maximum classification accuracy 84.62%) alone.

**Conclusions:**

Eye-tracking data from face-to-face conversations could accurately classify children with ASD and TD, suggesting that ASD might be objectively screened in everyday social interactions. However, these results will need to be validated with a larger sample of individuals with ASD (varying in severity and balanced sex ratio) using data collected from different modalities (eg, eye tracking, kinematic, electroencephalogram, and neuroimaging). In addition, individuals with other clinical conditions (eg, developmental delay and attention deficit hyperactivity disorder) should be included in similar ML studies for detecting ASD.

## Introduction

Autism spectrum disorder (ASD) is a complex neurodevelopmental condition characterized by social communication deficits along with restricted and repetitive behavior [1]. Owing to a lack of objective biomarkers, the current diagnosis of ASD heavily depends on behavioral evaluation, which involves substantive subjective procedures that can be negatively impacted by various factors such as caregivers’ reporting bias and clinicians’ insufficient capability in differentiating ASD [2,3]. In addition, the current diagnostic procedure is highly labor- and time-demanding due to the shortage in clinical specialists and requirement of lengthy examinations. A delayed diagnosis directly leads to postponed interventions, which subsequently impacts the prognosis of the affected children [4]. Therefore, seeking quantifiable and objective biomarkers of ASD, which could potentially make the diagnostic procedure more efficient and effective, has become a critical issue.

With respect to seeking objective biomarkers for ASD, recent studies reflect increasing interest in applying machine learning (ML) algorithms to examine whether features extracted from neuroimaging [5,6], electroencephalogram (EEG) [7], eye tracking [8,9], and kinematic data [10-12] could be used to identify ASD. The underlying justification for applying ML is based on the advantages of these approaches in identifying patterns that are not readily recognized by human eyes. Indeed, an ML approach demonstrated promising results in detecting ASD with objectively measured features. For example, Crippa et al [11] showed that seven kinematic features computed from a goal-directed motor task could accurately classify children with and without ASD (accuracy 96.7%). By implementing an imitation task, Li et al [13] reported a maximum classification accuracy of 86.7% using an ML approach.

Recently, a few studies have revealed that eye-tracking data could be used to identify ASD by implementing ML algorithms [8,9,14-19]. For example, Wan et al [9] recruited children within the ages of 4-6 years with ASD and typical development (TD) to watch a 10-second video displaying a woman speaking. ML features were extracted from eye-tracking measures in seven areas of interest (AOIs). Their results demonstrated that fixation time at the mouth and body AOIs could discriminate these two groups of participants with a classification accuracy of 85.1%. In contrast to Wan et al [9], who used a predefined AOI approach, Liu et al [8] used the *K*-means algorithm to extract features from the fixation data, which reached a maximum classification accuracy of 88.51%. Further, a few studies demonstrated that eye-tracking data obtained from web-searching tasks could be used to detect ASD [14-16]. Instead of computing features from eye-tracking data, Eraslan et al [15] performed a scan-path trend analysis to identify representative eye movement sequences for both individuals with ASD and TD. A classification was made based on the similarity of the individual’s visual scan path to the representative sequences. This approach was able to classify individuals with ASD and TD with above-chance accuracy.

The eye-tracking data used in these prior studies were primarily obtained by having participants watch images (ie, videos, pictures, web pages) [8,9,14]. However, in reality, human gaze behavior is highly context-sensitive. Existing findings show that experimental settings and cognitive load are critical factors that could influence how people visually attend [20,21]. In contrast to image-watching tasks, face-to-face interaction is a social task that is much more perceptually and cognitively difficult [22]. Other studies have shown that the presence of the social partner elicits a different pattern of both neural response and gaze behavior [23,24]. In this vein, findings obtained from image-viewing tasks could not be directly generalized to the scenario of natural social interaction. Accordingly, there is a need to investigate whether eye-tracking data from live social interaction could be used to identify ASD.

The major novelty of this study is that we investigated the feasibility of using eye-tracking data from face-to-face conversations to classify children with ASD and TD. This research question is of practical significance since face-to-face interaction is omnipresent in everyday life. With the development of eye-tracking technology that enables the detection of natural social gaze behavior, ASD might be initially screened in daily life without needing to undergo lengthy and sophisticated procedures in clinical settings. In addition, apart from visual fixation measures, we included the length of conversation as an input feature to investigate whether combining features from these two modalities would increase the classification performance. The majority of prior eye-tracking ML research focused on using gaze data to identify ASD. To the best of our knowledge, only two recent studies combined eye tracking and EEG or kinematic data, showing that combined features yielded better classification performance than using features from a single modality [19,25]. With the development of objective assessment, it is proposed that future detection of ASD might be realized by integrating data from different modalities. Our research therefore contributes to the existing literature by investigating whether combining data from visual fixation and length of conversation could improve the performance of ML models.

## Methods

### Participants

Data used in this study were obtained from a research project aiming at identifying behavioral markers of ASD. Twenty children with ASD and 23 children with TD were enrolled in the study. Children with ASD were recruited from the Child Psychiatry Department of Shenzhen Kangning Hospital. Owing to limited access to instruments such as the Autism Diagnostic Observation Schedule or the Autism Diagnostic Interview-Revised, ASD was primarily diagnosed by a licensed psychiatrist with no less than 5 years of clinical experience following the Diagnostic and Statistical Manual of Mental Disorders-IV criteria. In addition, the ASD diagnosis was further evaluated by a senior psychiatrist. A consultation with at least two additional senior psychiatrists would be arranged if there was disagreement among the specialists. All of these procedures ensured the correctness of the ASD diagnosis for the children enrolled in our study. Additional inclusion criteria were as follows: (1) aged between 6 and 13 years; (2) at least average nonverbal intelligence (IQ level was initially screened by the psychiatrist, and measured with the Raven advanced progressive matrices [26]); and (3) absence of other clinical conditions, including attention deficit hyperactivity disorder (ADHD) and schizophrenia. The TD group included healthy children without any mental or physical disorders and no diagnosis of ASD/ADHD in first-degree relatives, who were recruited from local schools. The experimental protocol followed the principles of the Declaration of Helsinki and the ethical guidelines of Shenzhen University. Written informed consent was provided by the participants’ caregivers.

### Data Collection

**Figure 1:**
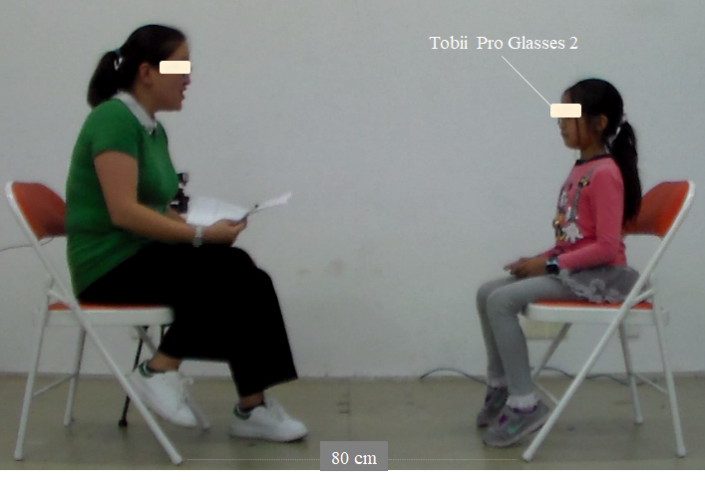
Experimental setup.

Participants were asked to engage in a structured face-to-face conversation with a 33-year-old female interviewer who was blinded to the participant’s group membership. The interviewer was required to behave consistently across all interviews with all participants. Participants were required to wear a head-mounted eye tracker (Tobii Pro Glasses 2; sampling rate: 50 Hz; Tobii Technology, Stockholm, Sweden) during the conversation, and they were seated 80 cm away from the interviewer’s chair (Figure 1). The conversation was videotaped with two still cameras. One camera (Samsung HMX-F90, sampling frequency 25 Hz) recorded both the interviewer and interviewee by placing each person equally on the left and right side of the recording view. The other camera (Logitech C270, sampling frequency 30Hz) was positioned beside the interviewer to capture the participant’s behavior from the front view.

Participants were not informed of the function of the eye tracker, and they were asked to avoid moving the glasses or to make any intense head movements during the conversation. A postexperiment interview confirmed that none of the participants was aware that their gaze behavior had been recorded. In addition, once the eye tracker was moved by the participant (particularly those with ASD), an accuracy test was performed at the end of the conversation to ensure the accuracy of the eye-tracking data recording. Verifications showed that Tobii Pro Glasses 2 was reliably accurate even if the glasses were moved by participants during the conversation.

The structured conversation consisted of four chronologically arranged sessions: general questions in the first session, hobby sharing in the second session, yes-no questions in the third session, and question raising in the fourth session. The first session allowed both the interviewer and the child to become familiarized with each other. The second session served the purpose of examining the participants’ behavior when speaking about their hobbies, which might induce different gaze behavior from that induced when discussing more generic topics [20]. The third session was designed to investigate the extent to which participants used head nodding or shaking to answer yes-no questions. The behavior of taking initiatives to raise questions was examined in the fourth session. Refer to Textbox 1 for further details of the questions used in each session.

**Details of the four sessions of the structured conversation.**

**Session 1: General questions**

1. What is your name?
2. How is your name written?
3. What is the name of your school and what grade are you in?
4. Who is your best friend? What is your favorite thing to do together?
5. Could you please share with me the most interesting thing that happened last week? Let me know the time, place, people, and the whole process of the event.
6. What is the plan for your summer vacation?

**Session 2: Hobby sharing**

1. What is your favorite thing to do? And can you tell me why you like doing it?

**Session 3: Yes-no questions**

1. Do you like apples?
2. Do you like to go to the zoo?
3. Do you like to go to school?
4. Do you like reading?
5. Do you like painting?
6. Do you like watching cartoons?
7. Do you like sports?
8. Do you like watching movies?
9. Do you like traveling?
10. Do you like shopping?

**Session 4: Question raising**

1. Now that I have asked you many questions, do you have any questions for me?

### Eye-Tracking Data Analysis

Data of four participants (one with ASD and three with TD) were discarded due to technical problems that occurred during the eye-tracking process. Hence, the final dataset consisted of 20 children with TD and 19 children with ASD. The participants’ demographic information is presented in Table 1.

The eye-tracking data were analyzed with Tobii Pro Lab software, which enables processing visual fixation data on dynamic stimuli. Note that the interviewer was also a dynamic stimulus as she was interacting with the participants throughout the conversation.

**Figure 2:**
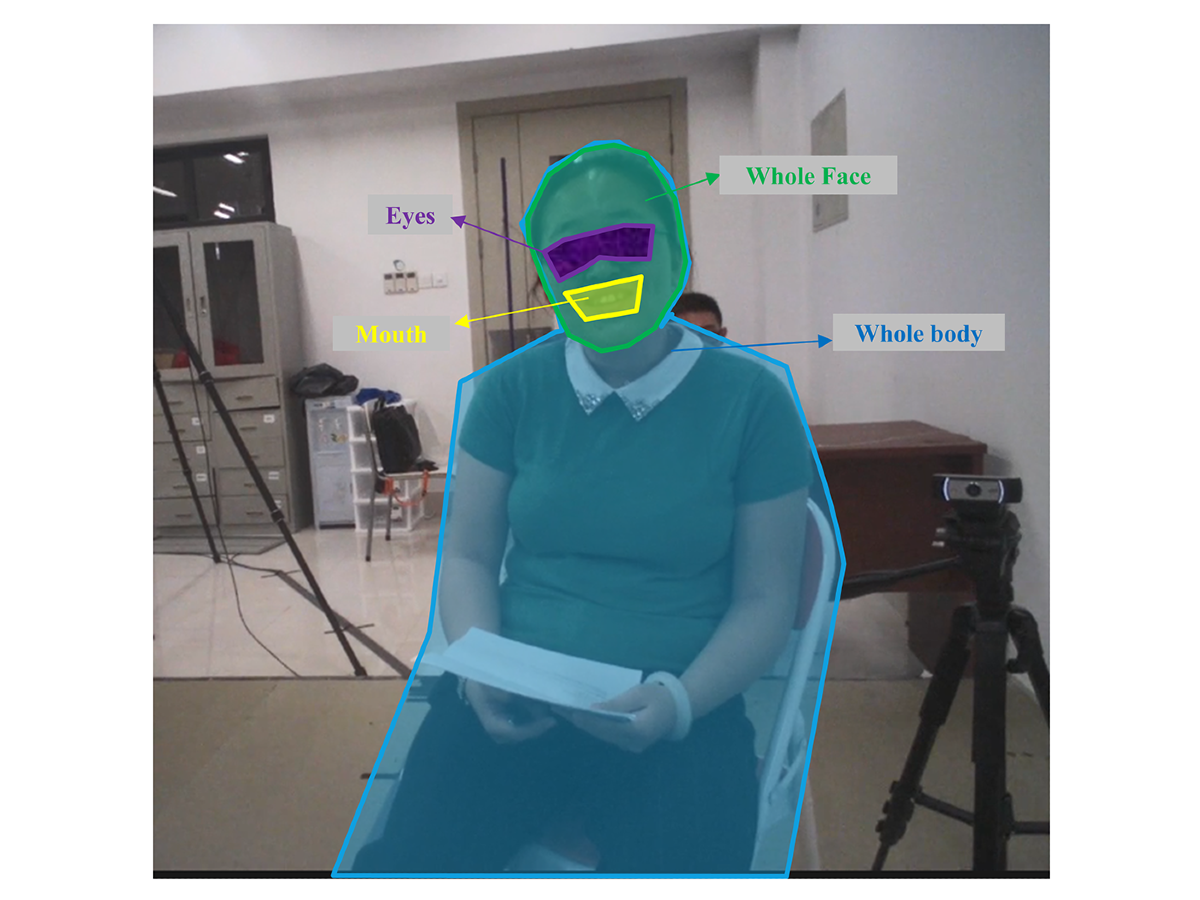
Four areas of interest.

Features were extracted on visual fixation and session length from the eye-tracking data. For the visual fixation features, four AOIs were analyzed, including the eyes, mouth, whole face, and whole body (Figure 2). We computed the percentage of visual fixation time on each AOI as features. Therefore, 16 AOI-based features were acquired (4 sessions × 4 AOIs).

**Table 1:** Comparison of demographic information and the area of interest (AOI)-based features in the autism spectrum disorder (ASD) and typical development (TD) groups.

Characteristic		ASD	TD	Group comparison	*P* value
**Demographic characteristics**					
	Sex ratio, M:F	17:2	17:3	χ^2^_1_=0.17	.68
	Age (months), mean (SD)	99.6 (25.1)	108.8 (27.0)	t_37_=1.09	.28
	IQ, mean (SD)	100.8 (22.7)	116.1 (22.7)	t_37_=2.45	.02
**AOI features, mean (SD)^a^**					
	Mouth_Session 1	0.05 (0.06)	0.19 (0.13)	*U*=59.5	<.001
	Eyes_Session 1	0.06 (0.06)	0.08 (0.09)	*U*=173.0	.63
	Face_Session 1	0.21 (0.17)	0.41 (0.18)	*U*=70.5	.001
	WholeBody_Session 1	0.33 (0.23)	0.55 (0.21)	*U*=85.5	.003
	Mouth_Session 2	0.05 (0.09)	0.16 (0.13)	*U*=88.0	.004
	Eyes_Session 2	0.04 (0.04)	0.06 (0.07)	*U*=143.0	.18
	Face_Session 2	0.17 (0.16)	0.39 (0.20)	*U*=77.0	.001
	WholeBody_Session 2	0.29 (0.26)	0.52 (0.25)	*U*=95.5	.008
	Mouth_Session 3	0.12 (0.15)	0.21 (0.17)	*U*=131.0	.10
	Eyes_Session 3	0.07 (0.06)	0.08 (0.10)	*U*=186.0	.91
	Face_Session 3	0.33 (0.26)	0.49 (0.21)	*U*=120.5	.05
	WholeBody_Session 3	0.46 (0.28)	0.06 (0.20)	*U*=134.5	.12
	Mouth_Session 4	0.05 (0.06)	0.12 (0.12)	*U*=122.0	.05
	Eyes_Session 4	0.06 (0.09)	0.08 (0.11)	*U*=183.5	.85
	Face_Session 4	0.21 (0.20)	0.32 (0.18)	*U*=120.0	.05
	WholeBody_Session 4	0.34 (0.25)	0.47 (0.22)	*U*=125.5	.07

^a^Due to a violation of the normality assumption, Mann-Whitney *U* tests were performed for group comparisons on AOI-based features.

To obtain the percentage of visual fixation time on each AOI, the first step was to draw a snapshot image from the eye-tracking video for the purpose of defining AOIs. Once AOIs were defined, with the help of the real-world mapping algorithm, Tobii Pro Lab automatically mapped the gaze point in the video onto correct spots of the snapshot image. The correctness of the mapping process was confirmed by a human observer. Manual mapping was performed in the case that no fixation was automatically mapped onto the snapshot or if the fixation automatically assigned failed to match the correct spot. In this way, the accuracy of visual fixation was reliably ensured. Note that we used the velocity-threshold identification fixation filter to define fixation, which meant that a fixation was detected if the velocity of the eye movement was below 30 degrees per second for no less than 60 milliseconds. Finally, the percentage of visual fixation time on each AOI in a session was computed as the length of the fixation time on the AOI divided by the total duration of the particular session. Results regarding the group comparison on the AOI-based features in different sessions are presented in Table 1.

The length of each session varied across participants. Mann-Whitney *U* tests showed that the children with ASD had significantly longer conversations in the first session (*U*=48, *P*<.01), second session (*U*=103, *P*=.02), and fourth session (*U*=107, *P*=.02), but not in the third session (*U*=150, *P*=.26). In addition, the total length of all four sessions was significantly longer in the ASD group (*U*=39, *P*<.01). These results indicated that session length might serve as an effective feature to classify children with ASD and TD. Thus, the lengths of the four sessions and the total session length were used as five input features.

### ML Procedure

#### Description of Dataset

Sixteen features on visual fixation (percentages of visual fixation time on four AOIs [mouth, eyes, face, and whole body] in four conversation sessions) and five features on session length were computed as features fed into the ML procedure. Therefore, the original dataset for the ML procedure was a 39 (participants)×21 (features) matrix. Three types of ML models were established, one with visual fixation features alone, one with session length features alone, and one with combined features on both modalities, to investigate whether combined features would yield better classification performance.

#### Classifiers

The classification task was performed by implementing four ML classifiers: support vector machine (SVM), linear discriminant analysis (LDA), decision tree (DT), and random forest (RF). The description of these classifiers is detailed below.

SVM is a supervised learning algorithm that has been previously implemented in classifying individuals with and without ASD [8,10]. The purpose of the SVM classifier is to create an optimal hyperplane in a multidimensional space with labeled training samples. Testing samples are classified based on the sign of the distance vector to the hyperplane, and the distance to the hyperplane determines the probability that they belong to the specific category.

The task of classifying children with ASD from those with TD is a binary classification problem. In this case, the LDA classifier works as a dimension reduction technique that projects all data points in the high-dimensional space onto a straight line (ie, one dimension) with training samples. Testing samples were classified in either group by the threshold value on the straight line.

The DT classifier is a tree-like flowchart. The nodes in the model represent tests on an attribute, the branches represent the outcomes of the tests, and the leaf nodes denote class labels. The DT classifier exhibits the advantage of strong interpretability, but it is prone to overfitting.

Instead of building a tree-like structure, the RF classifier is established by creating multiple simple trees with the training data. Test samples are categorized into a specific group based on the majority of votes from the trees.

#### Feature Selection

Forward feature selection (FFS) was applied to select features for model training and testing. Specifically, FFS is an iterative process starting with the evaluation of each individual feature by examining their classification performance. The feature with the highest classification accuracy would be preserved and is then combined with each of the other features to form two-feature models whose classification performances are further evaluated. The two features with optimal classification accuracy are then retained and used to establish three-feature models by combining them with each of the remaining features. By repeating these procedures, the one-feature, two-feature, …, *n*-feature models with the highest classification accuracy would be obtained (*n* represents the total number of examined features intended to be fed into ML models). In this way, FFS helped to identify not only the model with the highest classification accuracy but also the corresponding feature or feature combination.

#### Classification

**Figure 3:**
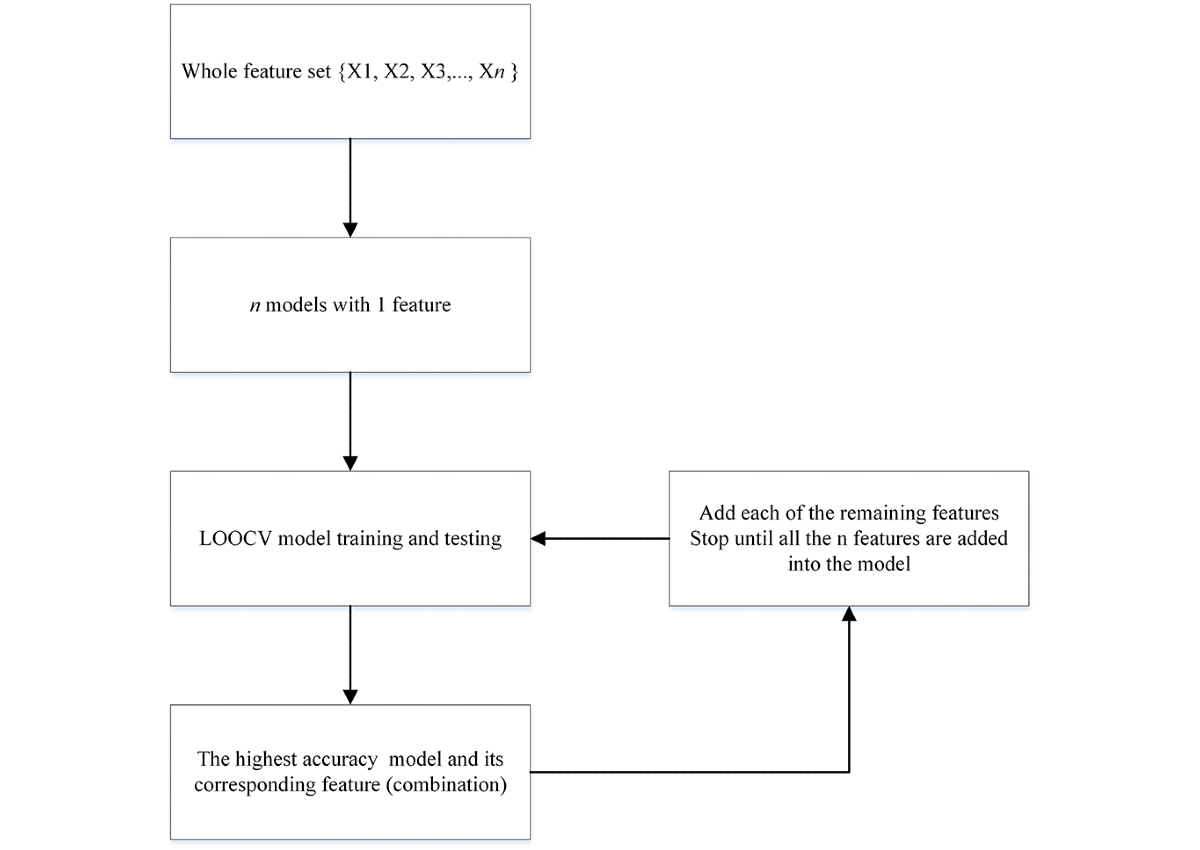
Flowchart of the machine learning procedure. LOOCV: leave-one-out cross-validation.

The entire ML procedure is schematically presented in Figure 3. To minimize the potential overfitting problem, we implemented leave-one-out cross-validation in ML model training and testing. Specifically, the test set contained only one participant sample and the remaining participant samples were used to train the ML model. This procedure was repeated until all participant samples were tested once. The accuracy, sensitivity, and specificity were computed to evaluate the classification of the ML models. Accuracy was defined as the percentage of participant samples that were correctly classified in both groups. Specificity and sensitivity corresponded to the model’s capability of correctly detecting the TD and ASD samples respectively.

## Results

### Classification with Combined Features

**Figure 4:**
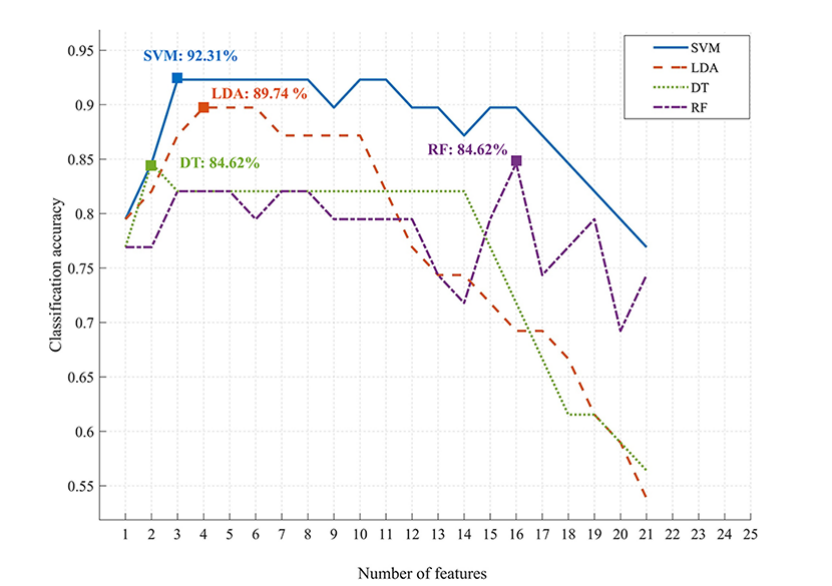
Variation of the classification accuracy with the number of features. SVM: support vector machine; LDA: linear discriminant analysis; DT: decision tree; RF: random forest.

The variation in classification accuracy according to the number of features used in the model is illustrated in Figure 4. All classifiers yielded a maximum classification accuracy above 84%. The SVM classifier achieved optimal classification accuracy of 92.31% with three features (specificity=100%, sensitivity=84.21%, area under the receiver operating characteristic curve [AUC]=0.92), followed by LDA with 89.74% accuracy using four features (specificity=90.00%, sensitivity=89.47%, AUC=0.92), DT with 84.62% accuracy using two features (specificity=80.00%, sensitivity=89.47%, AUC=0.86), and RF with 84.62% accuracy using 16 features (specificity=85.00%, sensitivity=84.21%, AUC=0.86).

The classification performance of the SVM classifier was the highest among the four classifiers. The variation of the SVM classification performance according to the number of features is presented in Table 2. The classification accuracy reached 79.49% with only one feature: total session length. The optimal classification accuracy of 92.31% was achieved with a minimum of three features: total session length, mouth in the first session, and whole body in the third session. 

**Table 2:** Variation of the support vector machine classification performance with different features.

Number of features	Added feature	Accuracy (%)	Sensitivity (%)	Specificity (%)
1	Total SL^a^	79.49	68.42	90.00
2	~^b^ +Mouth_Session 1	84.62	78.95	90.00
3	~ +Wholebody_Session 3	92.31	84.21	100.00
4	~ +Face_Session 3	92.31	84.21	100.00
5	~ +Face_Session 2	92.31	89.47	95.00
6	~ +Eyes_Session 4	92.31	89.47	95.00
7	~ +Face_Session 1	92.31	89.47	95.00
8	~ +SL_Session 2	92.31	89.47	95.00
9	~ +Wholebody_Session 1	89.74	89.47	90.00
10	~ +Face_Session 4	92.31	89.47	95.00
11	~ +Mouth_Session 2	92.31	89.47	95.00
12	~ +Eyes_Session 1	89.74	84.21	95.00
13	~ +Eyes_Session 2	89.74	84.21	95.00
14	~ +Mouth_Session 3	87.18	84.21	90.00
15	~ +SL_Session 3	89.74	84.21	95.00
16	~ +Wholebody_Session 4	89.74	84.21	95.00
17	~ +Mouth_Session 4	87.18	84.21	90.00
18	~ +Eyes_Session 3	84.62	78.95	90.00
19	~ +SL_Session 1	82.05	78.95	85.00
20	~ +SL_Session 4	79.49	78.95	80.00
21	~ +Wholebody_Session 2	76.92	73.68	80.00

^a^SL: session length.

^b^In forward feature selection, ~ represents all features in the previous iteration; for example, ~ represents all 6 previously selected features in the 7th iteration.

**Figure 5:**
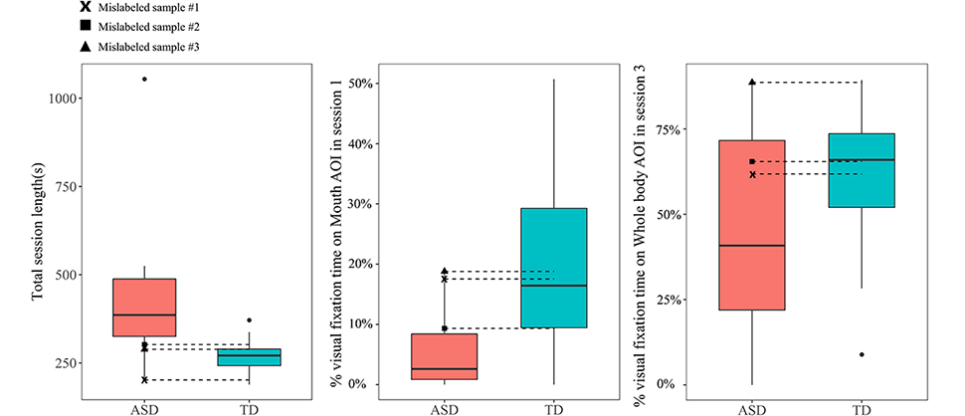
Boxplots of three features that achieved the highest classification accuracy in the support vector machine classifier along with the three mislabeled samples. ASD: autism spectrum disorder; TD: typical development.

The confusion matrix of this three-feature model that achieved the highest accuracy is presented in Table 3, which shows that the model correctly classified children in the TD group with 100% accuracy, but it mislabeled three children with ASD as having TD. Error analysis examining the mislabeled samples showed that these participants performed equally well as the children with TD (Figure 5). For example, the total session length of mislabeled sample 1 was shorter than that of 75% of the children in the TD group, and the visual fixation time on the mouth AOI in the first session was higher than that of half of the children in the TD group. Consistent with a previous study [27], these results support the significant heterogeneity among individuals with ASD.

**Table 3:** Confusion matrix of the support vector machine classifier with the highest accuracy.^a^

Actual class	Predicted class	
	TD^b^	ASD^c^
TD	TN^d^=20	FP^e^=0
ASD	FN^f^=3	TP^g^=16

^a^Accuracy=TP+TN/TP+FP+FN+TN; sensitivity=TP/TP+FN; specificity=TN/FP+TN.

^b^TD: typical development.

^c^ASD: autism spectrum disorder.

^d^TN: true negative.

^e^FP: false positive.

^f^FN: false negative.

^g^TP: true positive.

### Classification Using Only Visual Fixation Features

Following the same procedure but feeding only AOI-based features into the ML classifiers achieved a maximum classification accuracy of 84.62% by the LDA classifier (specificity=80.00%, sensitivity=89.47%, AUC=0.86) with three features (mouth in session 1, face in session 2, and mouth in session 3), and by the DT classifier (specificity=80.00%, sensitivity=89.47%, AUC=0.86) with two features (face in session 2 and eyes in session 3).

### Classification Using Only Session Length Features

When using only session length features to perform the classification task, the maximum classification accuracy of 84.62% was achieved by the SVM classifier (specificity=90.00%, sensitivity=78.95%, AUC=0.87) with four features (session length in sessions 1, 3, and 4, and total session length).

## Discussion

### Principal Findings

In this study, we extracted features on visual fixation and session length from eye-tracking data collected during face-to-face conversations and investigated their capacity for classifying children with ASD and TD. The maximum classification accuracy of 92.31% was achieved by combining features on both visual fixation and session length with the SVM classifier. The classification accuracy was higher than that obtained using visual fixation features (highest accuracy: 84.62%) or session length features (highest accuracy: 84.62%) alone. Since 19 children with ASD and 20 children with TD were enrolled in this study, there was a slight class imbalance. Majority class prediction is typically used as a baseline for imbalanced classification. In the context of this study, majority class prediction requires every participant sample to be predicted as “TD”. Thus, the classification accuracy of majority class prediction would be 51.3% (ie, 20/39), which is greatly lower than the optimal classification accuracy of our results. This suggests that our results could not be explained by majority class prediction.

The highest classification accuracy was achieved with three features: total session length, percentage of visual fixation time on the mouth AOI in the first session, and percentage of visual fixation time on the whole body AOI in the third session. As shown in Table 2, the total session length was an effective feature for discriminating ASD from TD with an accuracy of 79.49% alone. In our study, participants were engaged in a structured conversation, in which they had to interact with the interviewer by answering the same number of questions. Longer conversation might be explained by the social deficits in children with ASD. Specifically, it was assumed that children with ASD might have experienced greater difficulty in understanding the social information (eg, motivation, mental state, and emotion) conveyed by the interviewer [28,29]. Interestingly, various studies demonstrated that the social deficits are more pronounced when dealing with naturalistic social stimuli [29,30]. Thus, it took the children with ASD longer to finish the same number of questions. However, further exploration is needed to confirm whether the length of conversation could be attributed to the poor social understanding capacity.

Notably, fixation measures on the mouth and whole body AOIs played important roles in the SVM classifier that produced the highest classification accuracy. The mouth AOI emerged as a prominent feature in this study, possibly owing to the fact that participants were engaged in a conversational task. Previous studies showed that the mouth is an important body feature that affords the looking-toward behavior in conversations [22,31,32]. Our result of selecting the mouth AOI as an important feature was consistent with the findings of Wan et al [9], in which participants watched a video of a model speaking. With respect to the whole body AOI, abundant research has shown that individuals with ASD pay less attention to socially relevant stimuli [33,34]. The interviewer in this study could be viewed as the most relevant social stimulus, as participants needed to utilize information of the interviewer (eg, emotions, gestures, body movements) to converse with her. Looking away from the interviewer would induce the missing of important social information, which may further undermine the ability of the participants with ASD to interact with the interviewer during the conversation.

Apart from the fact that we used data from face-to-face interaction as opposed to data obtained from image-viewing tasks used in previous related studies, our study is different from other eye-tracking ML studies in two main aspects. First, this study recruited children aged between 6 and 13 years, whereas Wan et al [9] studied younger children (4-6 years old) and other studies [14-16,19] tested the adult population. Age is of profound significance in this context, since early identification and intervention may tremendously improve the prognosis of individuals with ASD [4]. A recent meta-analysis reported that the mean age at diagnosis of ASD was 60.48 months and was 43.18 months when only incorporating children aged ≤10 years [35]. This suggests that future ML studies should focus on examining younger children to facilitate the detection of ASD at an early stage. Second, the ASD severity level was not specifically measured in our study, which was accounted for in a previous study [27]. The children with ASD included in this study could be viewed as representing individuals with minor severity. It is recommended that individuals with ASD with different degrees of severity be included in future studies to improve the generalizability of the ML model. Except for these two differences, it is notable that our study and most others only classified individuals with ASD and TD [8,9,14-16]. Therefore, it remains unclear whether eye-tracking data could effectively detect ASD from other clinical phenotypes (eg, developmental delay and ADHD). More scientific endeavor is certainly required before a practical ML model that could detect ASD from different conditions is established.

### Limitations

To ensure that the participants would be able to converse with the interviewer, we recruited children within the age range of 6-13 years with at least average intellectual ability. Participants with severe symptoms of autism were not included. In addition, only four girls were enrolled in our study. Prior studies reported that males with ASD differ from females with ASD in many respects, including behavioral presentation, cognitive domains, and emotions [36,37]. Therefore, this study should only be considered as proof-of-concept research, which explored the feasibility of using eye-tracking data from face-to-face conversations to classify children with ASD and TD. Future studies might consider recruiting participants with various presentations (eg, different degrees of severity and balanced sex ratio) to ensure the generalizability of the ML model.

This study utilized a head-mounted eye tracker to record the gaze behavior, which might affect the social behavior of children with ASD to a larger extent. In general, individuals with ASD are more sensitive to wearing devices and eye-tracking techniques usually require extensive calibration [38,39]. These issues considerably raise the difficulty of implementing eye-tracking techniques on children with ASD, particularly on the younger population. To address these problems, a recent study used a webcam to record eye movement and developed a computer vision–based algorithm to detect gaze behavior. The results showed that the accuracy of the algorithm was comparable to that of manual coding when evaluating particular gaze behaviors [39]. It is proposed that more contactless and calibration-free techniques should be developed to record the gaze behavior in individuals with ASD.

Our study only computed the percentage of visual fixation time on different AOIs as measures of gaze behavior. In fact, a variety of other features could be obtained from the gaze behavior, including the number of fixations, entropy, and number of revisits [16,40]. Additionally, features extracted from oculomotor behavior are also recommended since atypical oculomotor performance has been extensively reported in individuals with ASD [41,42]. Future ML studies are encouraged to generate as many features as possible so as to allow for specification of the globally optimal set of features for ASD identification.

Using eye-tracking data from face-to-face interaction was a major novelty of this study. However, human interaction may introduce a variety of subjective factors that are difficult to control but might influence the gaze behavior of participants. For example, the interviewer might unconsciously behave differently with the children with ASD from the TD group, even if she was required to maintain a similar manner of behavior when interacting with participants in both groups. To examine whether the interviewer behaved consistently with both groups of participants, the overall amount of movement she made during the conversation was estimated using image differencing techniques applied to the video recordings [43,44]. Statistical analysis of these data showed that the amount of the interviewer’s movement was not significantly different when interacting with these two groups of participants (t_214_=1.76, *P*=.29). However, it is acknowledged that a similar amount of body movement does not necessarily mean that the interviewer’s behavior was completely identical for all participants. This is an inevitable problem faced by all studies investigating natural social interaction since no human being can be expected to behave exactly the same way when interacting with different people. In summary, future studies attempting to apply eye tracking to live social interactions need to cautiously control for factors (eg, context, task, and the interactant’s behavior) that might be introduced through human interaction. 

### Conclusion

Our study extracted features from eye-tracking data during face-to-face conversations to investigate their capacity of detecting children with ASD. With a relatively small sample, our results showed that combining features on visual fixation and session length could accurately classify children with ASD and those with TD. It is proposed that future eye-tracking ML studies could use features from gaze-based measures [8,9], visual scanning path [15], and oculomotor performance [41,42] to detect ASD. Finally, we recommend that a larger and younger participant sample should be tested with the ML approach by combining features obtained from different modalities (eye tracking, neuroimaging, EEG, and kinematic) to evaluate how these objectively measured features could contribute to the early screening of ASD.

## Abbreviations

ADHD: attention deficit hyperactivity disorder
AOI: area of interest
ASD: autism spectrum disorder
AUC: area under the receiver operating characteristic curve
DT: decision tree
EEG: electroencephalogram
FFS: forward feature selection
LDA: linear discriminant analysis
ML: machine learning
RF: random forest
SVM: support vector machine
TD: typical development
